# Penal Servitude and Insanity

**Published:** 1896-09-12

**Authors:** 


### Modern Sociology.

### PENAL SERVITUDE AND INSANITY.

An old question has recently been raised afresh, the mental condition of certain convicts having made people ask whether prison life is itself provocative of insanity.

The question is far more complex than at first sight might appear, and is just one of those on which a mere statistical answer would certainly lead to error.

There can be no doubt that a considerable proportion of the inmates of our prisons do become insane, and persons who know the gregarious nature of man's habits are not slow to say that when men are condemned to solitary confinement "that way madness lies." If, however, we grant that punishment of some sort is necessary for the suppression of crime, and if we also grant that the punishment to be inflicted must take the form of imprisonment rather than of corporal chastisement, the question resolves itself into this—what sort of imprisonment has the greatest deterrent effect upon the worst type of criminal, while at the same time producing the least possible injury to his body, his mind, and his moral nature? The question is not, and never can be, simply, Does imprisonment produce insanity? but rather, Does the form of imprisonment now in vogue tend more than any other possible form of imprisonment to have that effect?

From a careful consideration of the whole subject we feel assured that severe, terrible in fact, as is the English punishment of penal servitude, the modern modes of its infliction are not responsible for the insanity which unfortunately too often betrays itself in its course. To understand why so many convicts become, or prove to be, insane, it is essential to bear in mind what are the ordinarily recognised causes of insanity, even among those whose lives are untainted with any connection with crime, and how much more large a part these causes play in the lives of the criminal classes than in those of the common run of men. Among these causes are, first and foremost, heredity. Among our jail birds and habitual criminals, if there is one trait more striking than another it is the influence of heredity. On the one hand we find degenerate frames, rendering it difficult to them to compete with more sturdy specimens of the race. On the other hand, we find degenerate—perhaps undeveloped—nervous systems, leading them the more easily to yield to temptation, and making it the more difficult for them to bear the drudgery of honest labour, or to keep their unwilling muscles to the daily task of working for their daily bread. Both from bodily conformity and from mental instability, the hereditary members of the criminal classes, by whom our jails are so largely filled, are led to crime. We have, then, as the first factor in the undue preponderance of insanity among prisoners the fact that they are chiefly recruited from those very classes among whom the hereditary tendency to it is most marked.

The next great cause of insanity is drink, and no one who takes the trouble to study the character and the history of the prisoners who come before our police-courts and our criminal assizes will hesitate to admit the part played by drink in the etiology of crime. We do not here confine ourselves merely to the fact that a large amount of crime is committed while actually under the influence of alcohol; the point which for our present purpose it is important to insist upon is that outside and beyond the influence of alcoholic intoxication as a direct provocative to crime, the distress, the misery, the want, and the disease produced by habitual alcoholic excess drag men down into the ranks of those miserables upon whom the temptations to break the law are greatest, while the deterrent influence of imprisonment is least. Thus, again, prisoners are largely recruited from the ranks of those who are afflicted by the vice which of all others most tends to "steal men's wits away."

But if, without further considering the so-called predisposing causes of insanity, we turn to those conditions under which outbursts of mental disorder most commonly occur, we find prominent among them such causes as continued distress, prolonged anxiety, disappointment, indulgence in hatred and angry passions, and lack of occupation.

Again, except the last cause mentioned, are not all these conditions necessarily more incident upon those whose crime has been detected, whose schemes have been nipped, who have been betrayed by their companions, who (for even criminals have their virtues) know that during their incarceration their wives and little ones are thrown on an unkind world—are not, we say, all these well-recognised causes of insanity in far fuller operation among criminals who have been found out and are undergoing sentence, of whatever kind, than among the humdrum average population who lead their little uneventful lives in uneventful toil?

Quite irrespective, then, of the internal economy and discipline of our convict establishments, we must expect to find insanity among their inmates; and its presence, even in a larger degree than among those outside, is no necessary condemnation of their methods. So great, in fact, are the excesses, the excitement, and the irregularity of a life of crime that it is fair to believe that the regularity and order of a well-conducted convict establishment really saves more minds than it disturbs. The problem that is not yet solved is the provision of suitable mental occupation for the long weary years during which convicts are confined. The higher the type of mind the greater is the danger of unceasing introspection. Mere muscular exercise for the body is not hard to provide, but, except in regard to bodily health, it is not a complete success; occupation for the mind is a far greater difficulty. The discovery of useful occupations which shall at the same time employ the body, keep the mind from turning on itself, and train the prisoner in habits of self-supporting labour, is a most important problem, and so largely does the reformatory effect of our prison system depend upon it, that no trade influence should be allowed to interfere with the large development of prison industries. Steady work and the daily exercise of skill are far more reformatory than unthinking "labour" however "hard."

